# Altered cord blood mitochondrial DNA content and prenatal exposure to arsenic metabolites in low-arsenic areas

**DOI:** 10.21203/rs.3.rs-3414865/v1

**Published:** 2023-10-31

**Authors:** Feng Qiu, Hongling Zhang, Xin Wang, Zhenxian Jia, Yujie He, Yi Wu, Zhangpeng Li, Tongzhang Zheng, Wei Xia, Shunqing Xu, Yuanyuan Li

**Affiliations:** Huazhong University of Science and Technology Tongji Medical College; Wuchang University of Technology; Huazhong University of Science and Technology Tongji Medical College; Huazhong University of Science and Technology Tongji Medical College; Huazhong University of Science and Technology Tongji Medical College; Huazhong University of Science and Technology Tongji Medical College; Huazhong University of Science and Technology Tongji Medical College; Brown University; Huazhong University of Science and Technology Tongji Medical College; Huazhong University of Science and Technology Tongji Medical College; Tongji Medical College of Huazhong University of Science and Technology: Huazhong University of Science and Technology Tongji Medical College

**Keywords:** Arsenic Metabolism, Prenatal exposure, Mitochondrial DNA copy number, cord blood, Repeated measurement

## Abstract

While mitochondria are susceptible to environmental detriments, little is known about potential associations between arsenic metabolites and mitochondria DNA copy number (mtDNAcn). We attempted to examine whether arsenic metabolism in different trimesters was related to cord blood mtDNAcn alteration. We included 819 mother-newborn pairs embedded in an in-progress birth cohort survey performed from April 2014 to October 2016 in Wuhan, China. We determined maternal urinary arsenic species concentrations in different trimesters using HPLC-ICPMS. We decided on cord blood mtDNAcn using quantitative real-time polymerase chain reaction. In covariate-adjusted models, each two-fold increment of dimethylated arsenic (DMA) and total arsenic (TAs) in the 3rd trimester were related to 8.43% (95% CI: 1.13%, 16.26%) and 12.15% (95% CI:4.35%, 20.53%) increases in mtDNAcn, respectively. The dose-response trend with statistical significance was observed across tertiles of DMA and TAs in the 3rd trimester with mtDNAcn. These findings may prove the relationships between arsenic species and mitochondrial dysfunction.

## 1. Introduction

Arsenic widely occurs in nature, existing in organic and inorganic forms. Inorganic arsenic (iAs) is generally considered more toxic than organic forms. The World Health Organization has reported that iAs in the groundwater exists at high concentrations (> 10 μg/L) in many countries, including Bangladesh, China, India, Chile, and Mexico (Ravenscroft et al. 2009). Dietary intake has become an essential source of arsenic exposure because of its accumulation in rice, fish, and dairy products (Chain 2009). People from the USA and some European countries have been reported to be exposed to arsenic at levels up to 300 μg/day via food and beverages (European Food Safety 2014, Kurzius-Spencer et al. 2014). iAs could be methylated to organic forms recognized as monomethylated arsenic (MMA) and dimethylated arsenic (DMA). More highly methylated arsenicals could be excreted more readily through the urine, decreasing retention and decreased biological exposure, and toxicity (Vahter 2002). Nevertheless, the idea has received a challenge considering that methylated trivalent arsenic showed more toxicity than is (Stýblo et al. 2002).

Existing evidence has revealed arsenic contributes to adverse health outcomes (Ravenscroft et al. 2009). Long-term arsenic exposure could increase the hazards of cancer, diabetes, cardiovascular disease, and so on (ATSDR 2016, Kuo et al. 2017). Arsenic species could cross the placenta (Concha et al. 1998, Hall et al. 2007), resulting in newborns’ susceptibility to arsenic exposure (Farzan et al. 2013). Existing epidemiologic research has revealed that early life exposure to arsenic species was linked to unfavorable birth consequences [(e.g., low birth weight (LBW), preterm birth (PTB) (Gilbert-Diamond et al. 2016, Laine Jessica et al. 2015), poorer neurobehavioral performance and development of newborns (Chen et al. 2023, Liang et al. 2020, Soler-Blasco et al. 2022) ] and disease later in life (Chen et al. 2019, Steinmaus et al. 2016). Toxicological evidence demonstrates that exposure to inorganic (Huang et al. 2018, Rodriguez Karina et al. 2016, Rodriguez et al. 2020) and methylated arsenicals (Negro Silva Luis et al. 2017, Negro Silva Luis et al. 2021) could lead to adverse offspring health.

Mitochondria, an essential ingredient of eukaryotic cells, plays a prominent role in many biological processes, like reactive oxygen species (ROS) production, apoptosis, and lipid metabolism (Shadel &Horvath 2015, Wai &Langer 2016). Mitochondria’s vulnerability to environmental toxicants might be partially ascribed to its proximity to ROS (Dan Dunn et al. 2015), inadequate repair capacity, deficiency in noncoding introns, and lack of defensive histones (Malik &Czajka 2013). Nevertheless, mitochondria could compensate for genomic insults by modifying their abundance, ultimately altering mitochondrial DNA copy number (mtDNAcn) (Lee &Wei 2000). Generally, mtDNAcn, reflecting the number of copies of the mitochondrial genome per nucleated cell, is a hopeful marker of mitochondrial malfunction (Malik &Czajka 2013). Despite the variation in mtDNAcn among various cell types, mtDNAcn in specific tissues/cells could remain unchangeable due to its rigorous modulation. Accumulating epidemiological studies have associated unexpected mtDNAcn variation from oxidative stress or inflammation with chronic diseases (Hägg et al. 2021). Arsenic, widely distributed in nature, is recognized as hazardous to human health (Jomova &Valko 2011, Tian et al. 2023, Valko et al. 2005). Numerous epidemiological surveys have suggested that in-utero exposure to aluminum, manganese, and lead was related to elevated cord blood mtDNAcn (Kupsco et al. 2019, Liu et al. 2019, Smith Anna et al. 2021), while prenatal exposure to arsenic, magnesium, and thallium was linked to reduced cord blood mtDNAcn (Smith Anna et al. 2021, Song et al. 2020, Wu et al. 2019). Nevertheless, no published epidemiological surveys have investigated arsenic metabolism and mtDNAcn associations among susceptible populations (e.g., pregnant women and neonates).

We attempted to make up for earlier surveys by estimating the relationships between maternal urinary arsenic metabolites with cord blood mtDNAcn and confirming the crucial exposure windows.

## 2. Methods

### 2.1. Study population

The analysis population is a subset of the Wuhan Healthy Baby Cohort that has enrolled pregnancies at the first prenatal examination [gestational week less than 16] from 2012 to 2019. Eligibility criteria included fluency in Chinese, singleton pregnancy, and intent to reside in Wuhan throughout gestation. The present analysis was restricted to 819 mother-newborn pairs recruited between April 2014 and October 2016, with prenatal arsenic metabolism exposure data and neonatal mtDNAcn data available derived from earlier research (Wang et al. 2021).

Informed written consent was acquired from all participators at recruitment. The Huazhong University of Science Institutional review boards have authorized the research procedures.

### 2.2. Urine acquisition and arsenic metabolite quantification

Details of urinary arsenic metabolite quantification are depicted elsewhere (Wang et al. 2021). In short, midstream spot urine samples were gathered during scheduled antenatal visits and stored at −20°C. Arsenic species were quantified by isotope dilution high-performance liquid chromatography coupled with quadrupole inductively coupled plasma mass spectrometer (HPLC-ICPMS), including arsenobetaine (AsB), arsenite (As^3+^), arsenate (As^5+^), MMA and DMA. Information about quality assurance, including limits of detections (LODs), limits of quantifications (LOQs), and intra-day and inter-day coefficients of variation, are the same as that published by Wang et al.(Wang et al. 2021). Specific gravity (SG) was determined to standardize the urinary arsenic species levels.

### 2.3. Relative mtDNAcn measurement

Venous umbilical cord blood was collected in vacutainer tubes containing EDTA immediately after delivery. Before analysis, blood samples were reserved at −80°C. Relative mtDNAcn measurement was carried out according to a prior described method (Janssen Bram et al. 2012). Information about detailed procedures of quantitative real-time PCR is presented in the supporting materials.

### 2.4. Covariates

Maternal demographic data (e.g., maternal age, family annual income, education levels, passive smoking, and folic acid intake) were collected by trained personnel via semi-structured questionnaires. In addition, data on pregnancy (e.g., parity, neonate gender, and pregnancy complications) was retrieved from the medical records. Information about the classification of pre-pregnancy body mass index (BMI) is shown in Supplementary Methods. Secondhand smoke exposure throughout gestation was defined as passive smoke.

### 2.5. Statistical analysis

We conducted descriptive statistics analysis to characterize the distributions of variables of interest. Arsenic metabolite levels lower than the LOD were replaced with LOD/√2. We used SG to adjust for the dilution to correct urine dilution, which might potentially affect the association of arsenic metabolites with mtDNAcn. The formula of SG normalization is exhibited in the Supplementary Methods.

Urinary arsenic metabolite levels and relative mtDNAcn were log2-transformed to alleviate the skewed distribution. We calculated intraclass correlation coefficients (ICCs) to evaluate the reproducibility of urinary arsenic species levels in the 1st, 2nd, and 3rd trimesters (Rosner 2011). We averaged the SG-adjusted arsenic metabolite levels in different trimesters to generate individual-specific averages throughout gestation—arsenic metabolites. Details about arsenic exposure indices are shown in the Supplementary Methods.

Potential nonlinear associations between arsenic species and cord blood mtDNAcn were examined using 3-knot restricted cubic splines. Except for iAs and mtDNAcn (*P* = 0.008), linear relationships were observed between DMA, MMA, TAs, and mtDNAcn (*P* > 0.05). Thus, a general linear model was performed to examine the potential connection of individual-specific arsenic metabolite averages (continuous and in tertiles) across three trimesters with cord blood mtDNAcn. Also, generalized estimating equations (GEEs) were performed to assess the relationships of trimester-specific exposures (both continuous and in tertiles) with mtDNAcn (Sánchez Brisa et al. 2011). Tertile cut-points originated from trimester-specific determination, and we defined the lowest tertile as the reference. In addition, we modeled the median value of every tertile to determine the linear trends of these relationships. We converted beta (β) into percent change (%Δ) of exposures to make the statistical models more interpretable (Barrera-Gómez &Basagaña 2015). Covariates considered for inclusion were those changed estimates (≥ 10%) in the relationships or those observed to be linked to mtDNAcn in previous studies were based on previous studies and on. The following variables were included as covariables: maternal age at enrollment, gestational age (weeks), pre-pregnancy BMI (< 18.5, 18.5–23.9, ≥ 24 kg/m^2^), parity history (nulliparous or not), infant sex (male or not), education level (< 9, 9–12, ≥ 12 years), smoking exposure (passive smoking or not), gestational diabetes mellitus (yes or not), and gestational hypertension disorders (yes or not).

To test the robustness of our results, we completed additional analyses by excluding participants with PTB, LBW, gestational diabetes, or hypertensive disorders. Software SAS (version 9.4, Institute Inc., USA) was used.

## 3. Results

### 3.1. Characteristics

Table 1 exhibits the characteristics of the mother-newborn pairs. The mean maternal age at enrollment was 28.94 years. Approximately 68.74% of participating women had a normal pre-pregnancy weight, with an average pre-pregnancy BMI of 20.99 kg/m^2^. Of the mothers in the analysis sample, 27.11% of women reported exposure to secondhand smoke, 88.03% acquired a college education or above, and 80.59% were primiparous. The proportion of gestational diabetes and hypertension disorders was 8.06% and 2.93%, respectively. For newborns, 53.60% were male, and 3.42% were born preterm.

### 3.2. Distribution of maternal urinary arsenic parameters

Supplementary Table 1 exhibits the concentration profiles of the targets. The target analytes were detected with detection frequencies ranging from 64.2–98.7%. The median concentrations of maternal SG-normalized urinary DMA were 8.21, 7.72, and 7.93 μg/L, MMA was 1.10, 1.07, and 1.06 μg/L, iAs were 3.18, 3.66, and 3.82 μg/L, and the TAs were 13.58, 13.59, 13.49 μg/L in the 1st, 2nd and 3rd trimester, respectively. Throughout the pregnancy, the ICCs of SG-normalized arsenic metabolites varied from 0.17 to 0.39, indicating poor reproducibility of urinary arsenic species across pregnancy (Supplementary Table 2).

### 3.3. Relationships of maternal arsenic exposure with cord blood mtDNAcn

**Figure 1:**
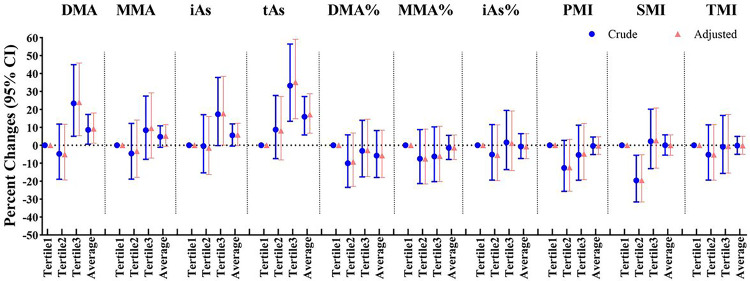
Associations of average maternal arsenic concentrations with cord blood mtDNAcn (N=819). Models were adjusted for maternal age at enrollment, gestational age, pre-pregnancy BMI, parity history, infant sex, education level, passive smoking during pregnancy, hypertensive disorders in pregnancy, and gestational diabetes mellitus.

Figure 1 lists the relationships of average levels of individual arsenic metabolites across three trimesters with the cord blood mtDNAcn alternation (Supplementary Table 3). In crude models, one double increase in DMA and TAs levels was related to an 8.56% (95% CI: 0.60, 17.16) and 15.94% (95% CI: 5.72, 27.14) increase in cord blood mtDNAcn, respectively. In adjusted models, the relationships of maternal DMA and TAs levels with cord blood mtDNAcn were still statistically significant (percent difference: 9.22%, 95% CI: 1.11, 17.98; percent difference: 17.21%, 95% CI: 6.70, 28.76). Additionally, the highest tertile of DMA concentrations was significantly linked to an approximately 23.97% (95% CI: 5.35, 45.89) increment in mtDNAcn compared with the lowest tertile. Moreover, a 35.14% increase of cord blood mtDNAcn was found in the highest tertile of TAs concentration, compared with the lowest tertile (percent difference: 35.14%, 95% CI: 14.80, 59.10). In the dose-response trend analysis, the positive association between TAs level and mtDNAcn remained significant (*p* trend = 0.0423), yet there wasn’t a remarkable relationship between DMA and mtDNAcn (*p* trend = 0.0854). Sensitivity analyses didn’t markedly alter our results (Supplementary Table 4).

**Figure 2:**
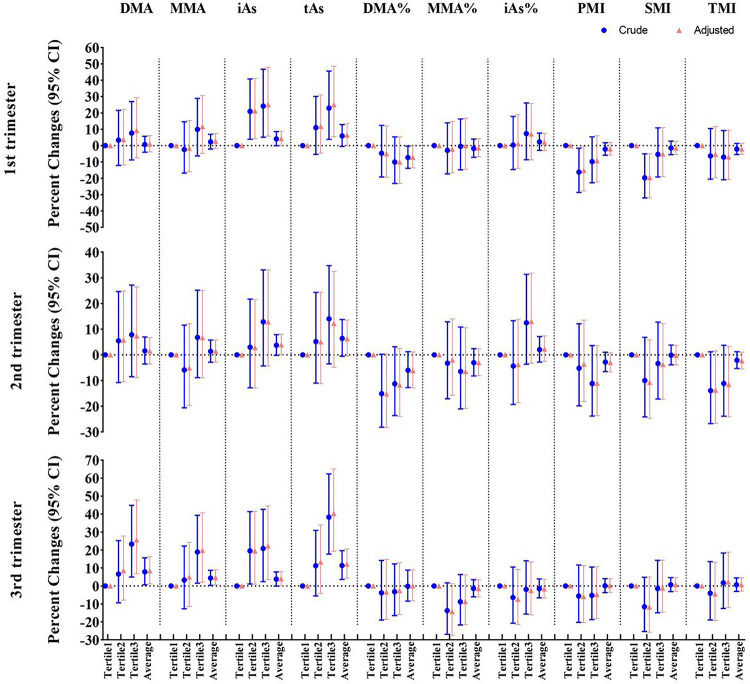
Trimester-specific associations between prenatal arsenic exposure and cord blood mtDNAcn. Models were adjusted for maternal age at enrollment, gestational age, pre-pregnancy BMI, parity history, infant sex, education level, passive smoking during pregnancy, hypertensive disorders in pregnancy, and gestational diabetes mellitus.

Figure 2 (Supplementary Table 5) exhibits the relationships of trimester-specific arsenic metabolite concentrations with cord blood mtDNAcn. In adjusted models, each two-fold increment of DMA, iAs, and TAs in the 3rd trimester was associated with 8.43% (95% CI: 1.13%, 16.26%), 3.94% (95% CI: 0.10%, 7.94%) and 12.15% (95% CI:4.35%, 20.53%) increases in mtDNAcn, respectively. Cord blood mtDNAcn increased across increasing tertiles of urinary DMA, iAs, and TAs levels in the 3rd trimester. The observed associations in sensitivity analyses didn’t change compared with the primary study results (Supplementary Table 6).

## 4. Discussion

In this longitudinal cohort survey, we evaluated the associations of maternal urinary arsenic species levels with cord blood mtDNAcn. We observed that elevated cord blood mtDNAcn was linked to exposure to DMA and TAs in the third trimester, where significant dose-response associations were also found.

Arsenic widely exists on the earth, leading to the risk of environmental exposure. It can be converted to MMA and DMA in humans, and urinary arsenic species are acknowledged as a marker of arsenic exposure (Hayakawa et al. 2005). The urinary arsenic speciation levels in this study were lower than those in Mexico (median of DMA, MMA, TAs were 20.6, 1.4, and 23.3 μg/L) (Laine Jessica et al. 2015) and Pabna (median of DMA, MMA, TAs were 50.2, 3.4 and 63.2 μg/L) pregnant women (Gao et al. 2019). Additionally, the urinary arsenic speciation levels in this study were higher than those of pregnant women in the INMA Project (geometric mean[GM] of DMA, MMA, iAs were 5.54, 0.28 and 0.27 μg/L) (Soler-Blasco et al. 2021), Oklahoma City (GM of DMA, MMA, iAs and TAs were 3.93, <LOD, 1.36 and 4.46 μg/L) (Chen et al. 2021), the PHIME-CROME project (GM of DMA, MMA, TAs were 2.67, 0.17 and 3.23 μg/L) (Stajnko et al. 2019) and the MADRES study (median of DMA, MMA, iAs and TAs were 4.4, 0.4, 0.5 and 5.4 μg/L) (Farzan et al. 2021). Different urinary arsenic speciation levels in other regions might result from variations in research participants, living environments, lifestyle behaviors, dietary intakes, and individual methylation efficiency. Urinary iAs reflect recently personal iAs absorbed dose, and unique iAs methylation patterns remain stable for 8–10 months (Steinmaus et al. 2005). Urinary arsenic concentration could be a surrogate for exogenous multiple exposure sources (Hughes Michael 2006, Kile Molly et al. 2009). Our reported ICCs (0.17–0.39) for repeated urinary arsenic speciation measurements were low, revealing high variability over the three trimesters. The low ICCs might be partly due to the alteration in external exposure level of arsenic speciation or the woman’s physiology attributed to the progression of pregnancy (Abduljalil et al. 2012). Given the poor reproducibility in this study, multiple or consecutive measurements along the pregnancy are desired to characterize arsenic speciation exposure during the whole pregnancy accurately.

The mtDNAcn, quantifying mitochondrial genome abundance, is a marker of mitochondrial function and oxidative stress (Castellani et al. 2020a, Malik &Czajka 2013). Mitochondria is the dominant cellular site of oxidative phosphorylation and ROS generation (Meyer et al. 2017). mtDNA is a prime victim of oxidative stress (Yakes &Van Houten 1997). mtDNAcn in most somatic cells ranges from 100 to 10,000 (Castellani et al. 2020b, Rausser et al. 2021). Mitochondria undergo fusion to complement mtDNA lesions via the transfer of nucleoids (Chen et al. 2010). Generally, the quantity of mtDNAcn within a cell is strictly governed to keep the cell in good condition (Attardi &Schatz 1988). Emerging epidemiology evidence has linked variation in mtDNAcn, either elevated or lower levels, to age-related disease (Castellani et al. 2020b, Hägg et al. 2021). Several epidemiological surveys have revealed that toxic exposures during early life were linked to changes in cord blood mtDNAcn. Increased cord blood mtDNAcn was found to be related to prenatal exposure to β-hexachlorocyclohexane (Vriens et al. 2017), aluminum (Liu et al. 2019), manganese (Kupsco et al. 2019), lead, and rare earth elements (gadolinium, dysprosium, erbium, and praseodymium) (Liu et al. 2020). Other surveys have shown that declined mtDNAcn was related to maternal smoking (Janssen et al. 2017), maternal lifetime stress (Brunst et al. 2017), prenatal exposure to thallium (Wu et al. 2019), arsenic (Song et al. 2020), magnesium (Smith Anna et al. 2021), air pollutants (PM_2.5_, PM_10_, and NO_2_) (Brunst et al. 2018, Clemente Diana et al. 2016, Hu et al. 2020, Janssen Bram et al. 2012), benzotriazoles (Chen et al. 2020). It’s comprehensible that the tendency of mtDNAcn changes varies from various pollutants. Additionally, findings for one exposure weren’t consistent. Therefore, current epidemiological surveys about the relationships of prenatal exposures with alterations in child mtDNAcn couldn’t draw firm agreements. This discordance in effects might be partly ascribed to the discrepancies in the characteristics of the study participants, types and levels of exposures, different tissues used to determine mtDNAcn (cord blood vs. placenta tissue), and duration of exposure. Limited research has examined the relationships of maternal arsenic metabolite concentrations with cord blood mtDNAcn.

In this study, we observed positive (urinary DMA and TAs) associations with cord blood mtDNAcn. Prior surveys have shown that people in arsenic-exposed areas have higher mtDNAcn (Ameer et al. 2016, Lou et al. 2022, Sanyal et al. 2018). To our knowledge, only one study in Belgium has shown that cord blood TAs was linked to an elevated placental mtDNAcn (Vriens et al. 2017). Increased mtDNAcn manifests enhanced mitochondrial biogenesis resulting in cell aberrant proliferation (Lee et al. 2011, Malik &Czajka 2013, Wei &Lee 2002). Previous experimental surveys have displayed that low-level arsenic exposure enhanced mitochondrial biogenesis (Lee et al. 2011, Lou et al. 2022). Lee et al. found that primary keratinocytes treated with arsenic at concentrations lower than 1.0 μmol/L exhibited increased cell proliferation and mitochondrial biogenesis (Lee et al. 2011). Luo et al. found mtDNAcn in Human hepatocyte L-02 cells treated with a low dose of iAs^3+^ (0.2 μmol/L) increased by more than 50% (Lou et al. 2022). Some surveys have reported that mtDNAcn increases with ROS to compensate for mtDNA lesions (Lee et al. 2000a, Liu et al. 2006, Wei et al. 2001). As a noted ROS stimulus, arsenic induces several types of ROS production, like peroxyl radicals, superoxide anion radicals, hydroxyl radicals, and hydrogen peroxide (Banerjee et al. 2011, Lynn et al. 2000). Little research has documented arsenite’s capability to promote mitochondrial ROS generation (Liu et al. 2005, Partridge et al. 2007). When ROS damaged mtDNA, reinforced mitochondrial biogenesis could increase mtDNAcn to make amends for mitochondrial malfunction (Giordano et al. 2014, Lee et al. 2000b). Nevertheless, excessive ROS might bring about declined mtDNA synthesis (Hou et al. 2010). Maintaining mtDNAcn in response to ROS induced by different pollutants remains unclear. Further surveys based on prospective cohorts and ingeniously-designed toxicological surveys are needed to elucidate the toxic mechanism for arsenic species. Our findings also showed a possibly enhanced susceptibility to arsenic species exposure in the 3rd trimester. Probable explanations might be variations in arsenic metabolite concentrations across pregnancy or chronic arsenic toxicity in mitochondria (Partridge et al. 2007). More studies are warranted to determine possible windows of prenatal exposure to arsenic species.

So far, this is the first survey on connections between maternal arsenic species and cord blood mtDNAcn, using repeated measurements. Notwithstanding, some disadvantages couldn’t be ignored. First, it wasn’t possible to eliminate the residual confounding by other unmeasured factors, like the lack of cell-type data for cord blood mtDNAcn. Blood samples used to quantify mtDNAcn consist of different cell types, and mtDNAcn in diverse immune cells vary (Hurtado-Roca et al. 2016). Equally, mtDNAcn is estimated according to genomic DNA originating from the buffy coat, which is confounded by both cell type distributions and contaminating platelets (Rausser et al. 2021). The lack of data on platelet count in the cord blood samples might result in the overestimation of leukocyte mtDNAcn quantification in consideration of high levels of mtDNA, not nuclear DNA, in platelets (Hurtado-Roca et al. 2016, Urata et al. 2008). Second, oxidative stress biomarkers in cord blood samples weren’t determined, which were associated with mitochondrial function (Liu et al. 2003). Finally, all participants in this survey were Chinese, which limited external generalizability to other populations. Further surveys of different people are suggested to confirm our findings.

## 5. Conclusion

In this prospective pre-birth cohort from Wuhan, China, DMA and TAs exposure in the third trimester was linked to elevated cord blood mtDNAcn. Our findings provided epidemiological data on mtDNA as a target of metals and emphasized a possible complicated part of arsenic specie in pathways of disease. Additional surveys are demanded to shed light on the molecular pathways involved in cord blood mtDNA damage induced by maternal methylated arsenic exposure.

## Figures and Tables

**Table 1:** Descriptive characteristics of study participants included in this study (N = 819).

Population characteristics	N (%) or mean ± SD
**Maternal age (years)**	28.94 ± 3.59
< 25	60 (7.33)
25–34	694 (84.74)
≥35	65 (7.94)
**Gestational week at labour (week)**	39.28 ± 1.15
**Pregnancy weight gain (kg)**	16.12 ± 4.72
< 10	56 (6.84)
10–15	273 (33.33)
16–19	308 (37.61)
> 20	182 (22.22)
**Pre-pregnancy BMI (kg/m^2^)**	20.99 ± 2.83
Underweight (< 18.5)	144 (17.58)
Normal (18.5–23.9)	563 (68.74)
Overweight (≥ 24)	112 (13.68)
**Parity**	
Primiparous	660 (80.59)
Multiparous	159 (19.41)
**Education (years)**	
≤ 9	46 (5.62)
9–12	98 (6.35)
>12	819 (88.03)
**Annual family income (yuan/year)**	
<50,000	88 (10.74)
50,000–99,999	290 (35.41)
≥100000	437 (53.36)
Missing	4 (0.49)
**Passive smoke during pregnancy**	
No	595 (72.65)
Yes	222 (27.11)
Missing	2 (0.24)
**Gestational diabetes mellitus**	
No	753 (91.94)
Yes	66 (8.06)
**Gestational hypertension disorder**	
No	795 (97.07)
Yes	24 (2.93)
**Iron supplementation during pregnancy**	
No	424 (51.77)
Yes	395 (48.23)
**Folic acid supplementation during pregnancy**	
No	110 (13.43)
Yes	709 (86.57)
**Fetal sex**	
Male	439 (53.60)
Female	380 (46.40)
**Preterm birth**	
Yes	28 (3.42)
No	791 (96.58)
**Birth weight (g)**	3339.87 ± 430.87
**Cord blood mtDNAcn** ^a^	0.92 (1.95)

Abbreviations: SD, standard deviance; N, number; %, proportion; BMI, body mass index; mtDNAcn, mitochondrial DNA copy number.

a Geometric mean (geometric standard deviation).

## Data Availability

The datasets analyzed during the current study are available from the corresponding author on reasonable request.

## References

[1] Abduljalil K, Furness P, Johnson TN, Rostami-Hodjegan A, Soltani H (2012): Anatomical, Physiological and Metabolic Changes with Gestational Age during Normal Pregnancy. Clin Pharmacokinet 51, 365–396 22515555 10.2165/11597440-000000000-00000

[2] Ameer SS, Xu Y, Engström K, Li H, Tallving P, Nermell B, Boemo A, Parada LA, Peñaloza LG, Concha G, Harari F, Vahter M, Broberg K (2016): Exposure to Inorganic Arsenic Is Associated with Increased Mitochondrial DNA Copy Number and Longer Telomere Length in Peripheral Blood. Front Cell Dev Biol 4 27597942 10.3389/fcell.2016.00087 PMC4992680

[3] ATSDR 2016: Toxicological profile for arsenic U.S. Department of Health and Human Services, Public Health Service, Atlanta, GA

[4] Attardi G, Schatz G (1988): Biogenesis of Mitochondria. Annu Rev Cell Biol 4, 289–331 2461720 10.1146/annurev.cb.04.110188.001445

[5] Banerjee M, Bhattacharjee P, Giri AK (2011): Arsenic-induced Cancers: A Review with Special Reference to Gene, Environment and Their Interaction. Genes and Environment 33, 128–140

[6] Barrera-Gómez J, Basagaña X (2015): Models with Transformed Variables: Interpretation and Software. Epidemiology 26, e16–e17 25643111 10.1097/EDE.0000000000000247

[7] Brunst KJ, Sanchez Guerra M, Gennings C, Hacker M, Jara C, Bosquet Enlow M, Wright RO, Baccarelli A, Wright RJ (2017): Maternal Lifetime Stress and Prenatal Psychological Functioning and Decreased Placental Mitochondrial DNA Copy Number in the PRISM Study. Am J Epidemiol 186, 1227–1236 28595325 10.1093/aje/kwx183 PMC5859981

[8] Brunst KJ, Sanchez-Guerra M, Chiu Y-HM, Wilson A, Coull BA, Kloog I, Schwartz J, Brennan KJ, Bosquet Enlow M, Wright RO, Baccarelli AA, Wright RJ (2018): Prenatal particulate matter exposure and mitochondrial dysfunction at the maternal-fetal interface: Effect modification by maternal lifetime trauma and child sex. Environ Int 112, 49–58 29248865 10.1016/j.envint.2017.12.020 PMC6094933

[9] Castellani CA (2020a): Mitochondrial DNA copy number can influence mortality and cardiovascular disease via methylation of nuclear DNA CpGs. Genome Med 12, 84 32988399 10.1186/s13073-020-00778-7 PMC7523322

[10] Castellani CA, Longchamps RJ, Sun J, Guallar E, Arking DE (2020b): Thinking outside the nucleus: Mitochondrial DNA copy number in health and disease. Mitochondrion 53, 214–223 32544465 10.1016/j.mito.2020.06.004 PMC7375936

[11] Chain EPoCitF (2009): Scientific Opinion on Arsenic in Food. EFSA Journal 7, 1351

[12] Chen H, Vermulst M, Wang YE, Chomyn A, Prolla TA, McCaffery JM, Chan DC (2010): Mitochondrial Fusion Is Required for mtDNA Stability in Skeletal Muscle and Tolerance of mtDNA Mutations. Cell 141, 280–289 20403324 10.1016/j.cell.2010.02.026 PMC2876819

[13] Chen H, Zhang H, Wang X, Wu Y, Zhang Y, Chen S, Zhang W, Sun X, Zheng T, Xia W, Xu S, Li Y (2023): Prenatal arsenic exposure, arsenic metabolism and neurocognitive development of 2-year-old children in low-arsenic areas. Environ Int 174, 107918 37043832 10.1016/j.envint.2023.107918

[14] Chen W-J, Davis EM, Stoner JA, Robledo C, Goodman JR, Garwe T, Janitz AE, Xu C, Hwang J, Peck JD (2021): Urinary total arsenic and arsenic methylation capacity in pregnancy and gestational diabetes mellitus: A case-control study. Chemosphere 271, 129828 33736216 10.1016/j.chemosphere.2021.129828 PMC8966639

[15] Chen X, Zhou Y, Hu C, Xia W, Xu S, Cai Z, Li Y (2020): Prenatal exposure to benzotriazoles and benzothiazoles and cord blood mitochondrial DNA copy number: A prospective investigation. Environ Int 143, 105920 32653801 10.1016/j.envint.2020.105920

[16] Chen Y, Wu F, Liu X, Parvez F, LoIacono NJ, Gibson EA, Kioumourtzoglou M-A, Levy D, Shahriar H, Uddin MN, Islam T, Lomax A, Saxena R, Sanchez T, Santiago D, Ellis T, Ahsan H, Wasserman GA, Graziano JH (2019): Early life and adolescent arsenic exposure from drinking water and blood pressure in adolescence. Environ Res 178, 108681 31520830 10.1016/j.envres.2019.108681 PMC7010462

[17] Clemente Diana BP (2016): Prenatal Ambient Air Pollution, Placental Mitochondrial DNA Content, and Birth Weight in the INMA (Spain) and ENVIRONAGE (Belgium) Birth Cohorts. Environ Health Perspect 124, 659–665 26317635 10.1289/ehp.1408981 PMC4858384

[18] Concha G, Vogler G, Fau - Lezcano D, Lezcano D, Fau - Nermell B, Nermell B, Fau - Vahter M, Vahter M (1998): Exposure to inorganic arsenic metabolites during early human development. Toxicol Sci 44, 185–190 9742656 10.1006/toxs.1998.2486

[19] Dan Dunn J, Alvarez LAJ, Zhang X, Soldati T (2015): Reactive oxygen species and mitochondria: A nexus of cellular homeostasis. Redox Biol 6, 472–485 26432659 10.1016/j.redox.2015.09.005 PMC4596921

[20] European Food Safety A (2014): Dietary exposure to inorganic arsenic in the European population. EFSA Journal 12, 3597 10.2903/j.efsa.2021.6380 PMC7845508 33537067

[21] Farzan SF, Karagas MR, Chen Y (2013): In utero and early life arsenic exposure in relation to long-term health and disease. Toxicol Appl Pharmacol 272, 384–390 23859881 10.1016/j.taap.2013.06.030 PMC3783578

[22] Farzan SF, Howe CG, Chavez TA, Hodes TL, Johnston JE, Habre R, Dunton G, Bastain TM, Breton CV (2021): Demographic predictors of urinary arsenic in a low-income predominantly Hispanic pregnancy cohort in Los Angeles. J Expo Sci Environ Epidemiol 31, 94–107 32719440 10.1038/s41370-020-0251-1 PMC7796897

[23] Gao S, Lin P-I, Mostofa G, Quamruzzaman Q, Rahman M, Rahman ML, Su L, Hsueh Y-m, Weisskopf M, Coull B, Christiani DC (2019): Determinants of arsenic methylation efficiency and urinary arsenic level in pregnant women in Bangladesh. Environ Health 18, 94 31690343 10.1186/s12940-019-0530-2 PMC6833186

[24] Gilbert-Diamond D, Emond Jennifer A, Baker Emily R, Korrick Susan A, Karagas Margaret R (2016): Relation between in Utero Arsenic Exposure and Birth Outcomes in a Cohort of Mothers and Their Newborns from New Hampshire. Environ Health Perspect 124, 1299–1307 26955061 10.1289/ehp.1510065 PMC4977046

[25] Giordano C (2014): Efficient mitochondrial biogenesis drives incomplete penetrance in Leber’s hereditary optic neuropathy. Brain 137, 335–353 24369379 10.1093/brain/awt343 PMC3914475

[26] Hägg S, Jylhävä J, Wang Y, Czene K, Grassmann F (2021): Deciphering the genetic and epidemiological landscape of mitochondrial DNA abundance. Hum Genet 140, 849–861 33385171 10.1007/s00439-020-02249-w PMC8099832

[27] Hall M, Gamble M, Slavkovich V, Liu X, Levy D, Cheng Z, van Geen A, Yunus M, Rahman M, Pilsner JR, Graziano J (2007): Determinants of Arsenic Metabolism: Blood Arsenic Metabolites, Plasma Folate, Cobalamin, and Homocysteine Concentrations in Maternal–Newborn Pairs. Environ Health Perspect 115, 1503–1509 17938743 10.1289/ehp.9906 PMC2022678

[28] Hayakawa T, Kobayashi Y, Cui X, Hirano S (2005): A new metabolic pathway of arsenite: arsenic–glutathione complexes are substrates for human arsenic methyltransferase Cyt19. Arch Toxicol 79, 183–191 15526190 10.1007/s00204-004-0620-x

[29] Hou L, Zhu Z-Z, Zhang X, Nordio F, Bonzini M, Schwartz J, Hoxha M, Dioni L, Marinelli B, Pegoraro V, Apostoli P, Bertazzi PA, Baccarelli A (2010): Airborne particulate matter and mitochondrial damage: a cross-sectional study. Environ Health 9, 48 20696069 10.1186/1476-069X-9-48 PMC2928195

[30] Hu C, Sheng X, Li Y, Xia W, Zhang B, Chen X, Xing Y, Li X, Liu H, Sun X, Xu S (2020): Effects of prenatal exposure to particulate air pollution on newborn mitochondrial DNA copy number. Chemosphere 253, 126592 32289600 10.1016/j.chemosphere.2020.126592

[31] Huang MC, Douillet C, Dover EN, Stýblo M (2018): Prenatal arsenic exposure and dietary folate and methylcobalamin supplementation alter the metabolic phenotype of C57BL/6J mice in a sex-specific manner. Arch Toxicol 92, 1925–1937 29721587 10.1007/s00204-018-2206-z PMC6611168

[32] Hughes Michael F (2006): Biomarkers of Exposure: A Case Study with Inorganic Arsenic. Environ Health Perspect 114, 1790–1796 17107869 10.1289/ehp.9058 PMC1665401

[33] Hurtado-Roca Y, Ledesma M, Gonzalez-Lazaro M, Moreno-Loshuertos R, Fernandez-Silva P, Enriquez JA, Laclaustra M (2016): Adjusting MtDNA Quantification in Whole Blood for Peripheral Blood Platelet and Leukocyte Counts. PLOS ONE 11, e0163770 27736919 10.1371/journal.pone.0163770 PMC5063275

[34] Janssen BG, Gyselaers W, Byun H-M, Roels HA, Cuypers A, Baccarelli AA, Nawrot TS (2017): Placental mitochondrial DNA and CYP1A1 gene methylation as molecular signatures for tobacco smoke exposure in pregnant women and the relevance for birth weight. J Transl Med 15, 5 28052772 10.1186/s12967-016-1113-4 PMC5209876

[35] Janssen Bram G, Munters E, Pieters N, Smeets K, Cox B, Cuypers A, Fierens F, Penders J, Vangronsveld J, Gyselaers W, Nawrot Tim S (2012): Placental Mitochondrial DNA Content and Particulate Air Pollution during in Utero Life. Environ Health Perspect 120, 1346–1352 22626541 10.1289/ehp.1104458 PMC3440109

[36] Jomova K, Valko M (2011): Advances in metal-induced oxidative stress and human disease. Toxicology 283, 65–87 21414382 10.1016/j.tox.2011.03.001

[37] Kile Molly L, Hoffman E, Hsueh Y-M, Afroz S, Quamruzzaman Q, Rahman M, Mahiuddin G, Ryan L, Christiani David C (2009): Variability in Biomarkers of Arsenic Exposure and Metabolism in Adults over Time. Environ Health Perspect 117, 455–460 19337522 10.1289/ehp.11251 PMC2661917

[38] Kuo C-C, Moon Katherine A, Wang S-L, Silbergeld E, Navas-Acien A (2017): The Association of Arsenic Metabolism with Cancer, Cardiovascular Disease, and Diabetes: A Systematic Review of the Epidemiological Evidence. Environ Health Perspect 125, 087001 28796632 10.1289/EHP577 PMC5880251

[39] Kupsco A, Sanchez-Guerra M, Amarasiriwardena C, Brennan KJM, Estrada-Gutierrez G, Svensson K, Schnaas L, Pantic I, Téllez-Rojo MM, Baccarelli AA, Wright RO (2019): Prenatal manganese and cord blood mitochondrial DNA copy number: Effect modification by maternal anemic status. Environ Int 126, 484–493 30849576 10.1016/j.envint.2019.02.029 PMC6471611

[40] Kurzius-Spencer M, Burgess JL, Harris RB, Hartz V, Roberge J, Huang S, Hsu C-H, O’Rourke MK (2014): Contribution of diet to aggregate arsenic exposures—An analysis across populations. J Expo Sci Environ Epidemiol 24, 156–162 23860400 10.1038/jes.2013.37 PMC4027043

[41] Laine Jessica E, Bailey Kathryn A, Rubio-Andrade M, Olshan Andrew F, Smeester L, Drobná Z, Herring Amy H, Stýblo M, García-Vargas Gonzalo G, Fry Rebecca C (2015): Maternal Arsenic Exposure, Arsenic Methylation Efficiency, and Birth Outcomes in the Biomarkers of Exposure to ARsenic (BEAR) Pregnancy Cohort in Mexico. Environ Health Perspect 123, 186–192 25325819 10.1289/ehp.1307476 PMC4314242

[42] Lee C-H, Wu S-B, Hong C-H, Liao W-T, Wu C-Y, Chen G-S, Wei Y-H, Yu H-S (2011): Aberrant Cell Proliferation by Enhanced Mitochondrial Biogenesis via mtTFA in Arsenical Skin Cancers. Am J Pathol 178, 2066–2076 21514422 10.1016/j.ajpath.2011.01.056 PMC3081159

[43] Lee H-C, Wei Y-H (2000): Mitochondrial role in life and death of the cell. J Biomed Sci 7, 2–15 10644884 10.1007/BF02255913

[44] Lee HC, Ph Yin, Fau - Lu CY, Lu Cy, Fau - Chi CW, Chi Cw, Fau - Wei YH, Wei YH (2000a): Increase of mitochondria and mitochondrial DNA in response to oxidative stress in human cells. PMC1221082 10816438

[45] Lee HC, Yin Ph, Fau - Lu CY, Lu Cy, Fau - Chi CW, Chi Cw, Fau - Wei YH, YH Wei (2000b): Increase of mitochondria and mitochondrial DNA in response to oxidative stress in human cells. Biochem J 348, 425–432 10816438 PMC1221082

[46] Liang C, Wu X, Huang K, Yan S, Li Z, Xia X, Pan W, Sheng J, Tao R, Tao Y, Xiang H, Hao J, Wang Q, Tong S, Tao F (2020): Domain- and sex-specific effects of prenatal exposure to low levels of arsenic on children’s development at 6 months of age: Findings from the Ma’anshan birth cohort study in China. Environ Int 135, 105112 31881426 10.1016/j.envint.2019.105112

[47] Liu B, Song L, Zhang L, Wu M, Wang L, Cao Z, Zhang B, Xu S, Wang Y (2019): Prenatal aluminum exposure is associated with increased newborn mitochondrial DNA copy number. Environ Pollut 252, 330–335 31158661 10.1016/j.envpol.2019.05.116

[48] Liu C-S, Tsai C-S, Kuo C-L, Chen H-W, Lii C-K, Ma Y-S, Wei Y-H (2003): Oxidative Stress-related Alteration of the Copy Number of Mitochondrial DNA in Human Leukocytes. Free Radic Res 37, 1307–1317 14753755 10.1080/10715760310001621342

[49] Liu CS, Cheng WL, Lee CF, Ma YS, Lin CY, Huang CC, Wei YH (2006): Alteration in the copy number of mitochondrial DNA in leukocytes of patients with mitochondrial encephalomyopathies. Acta Neurol Scand 113, 334–341 16629770 10.1111/j.1600-0404.2006.00586.x

[50] Liu S-X, Davidson MM, Tang X, Walker WF, Athar M, Ivanov V, Hei TK (2005): Mitochondrial Damage Mediates Genotoxicity of Arsenic in Mammalian Cells. Cancer Res 65, 3236–3242 15833855 10.1158/0008-5472.CAN-05-0424

[51] Liu Y, Wu M, Liu B, Song L, Bi J, Wang L, Upadhyaya Khatiwada S, Chen K, Liu Q, Xiong C, Li Y, Xia W, Xu S, Wang Y, Zhou A (2020): Association of prenatal exposure to rare earth elements with newborn mitochondrial DNA content: Results from a birth cohort study. Environ Int 143, 105863 32683209 10.1016/j.envint.2020.105863

[52] Lou Q, Zhang M, Zhang K, Liu X, Zhang Z, Zhang X, Yang Y, Gao Y (2022): Arsenic exposure elevated ROS promotes energy metabolic reprogramming with enhanced AKT-dependent HK2 expression. Sci Total Environ 836, 155691 35525345 10.1016/j.scitotenv.2022.155691

[53] Lynn S, Gurr J-R, Lai H-T, Jan K-Y (2000): NADH Oxidase Activation Is Involved in Arsenite-Induced Oxidative DNA Damage in Human Vascular Smooth Muscle Cells. Circ Res 86, 514–519 10720412 10.1161/01.res.86.5.514

[54] Malik AN, Czajka A (2013): Is mitochondrial DNA content a potential biomarker of mitochondrial dysfunction? Mitochondrion 13, 481–492 23085537 10.1016/j.mito.2012.10.011

[55] Meyer JN, Leuthner TC, Luz AL (2017): Mitochondrial fusion, fission, and mitochondrial toxicity. Toxicology 391, 42–53 28789970 10.1016/j.tox.2017.07.019 PMC5681418

[56] Negro Silva Luis F, Lemaire M, Lemarié Catherine A, Plourde D, Bolt Alicia M, Chiavatti C, Bohle DS, Slavkovich V, Graziano Joseph H, Lehoux S, Mann Koren K (2017): Effects of Inorganic Arsenic, Methylated Arsenicals, and Arsenobetaine on Atherosclerosis in the apoE−/− Mouse Model and the Role of As3mt-Mediated Methylation. Environ Health Perspect 125, 077001 28728140 10.1289/EHP806 PMC5744679

[57] Negro Silva Luis F, Makhani K, Lemaire M, Lemarié Catherine A, Plourde D, Bolt Alicia M, Chiavatti C, Bohle DS, Lehoux S, Goldberg Mark S, Mann Koren K (2021): Sex-Specific Effects of Prenatal and Early Life Inorganic and Methylated Arsenic Exposure on Atherosclerotic Plaque Development and Composition in Adult ApoE−/− Mice. Environ Health Perspect 129, 057008 34014776 10.1289/EHP8171 PMC8136521

[58] Partridge MA, Huang SXL, Hernandez-Rosa E, Davidson MM, Hei TK (2007): Arsenic Induced Mitochondrial DNA Damage and Altered Mitochondrial Oxidative Function: Implications for Genotoxic Mechanisms in Mammalian Cells. Cancer Res 67, 5239–5247 17545603 10.1158/0008-5472.CAN-07-0074

[59] Rausser S, Trumpff C, McGill MA, Junker A, Wang W, Ho S-H, Mitchell A, Karan KR, Monk C, Segerstrom SC, Reed RG, Picard M (2021): Mitochondrial phenotypes in purified human immune cell subtypes and cell mixtures. eLife 10, e70899 34698636 10.7554/eLife.70899 PMC8612706

[60] Ravenscroft P, Brammer H, Richards K (2009): Arsenic Pollution: A Global Synthesis. RGS-IBG Book Series 1

[61] Rodriguez Karina F, Ungewitter Erica K, Crespo-Mejias Y, Liu C, Nicol B, Kissling Grace E, Yao Humphrey H-C (2016): Effects of in Utero Exposure to Arsenic during the Second Half of Gestation on Reproductive End Points and Metabolic Parameters in Female CD-1 Mice. Environ Health Perspect 124, 336–343 26295903 10.1289/ehp.1509703 PMC4786990

[62] Rodriguez KF, Mellouk N, Ungewitter EK, Nicol B, Liu C, Brown PR, Willson CJ, Yao HHC (2020): In utero exposure to arsenite contributes to metabolic and reproductive dysfunction in male offspring of CD-1 mice. Reprod Toxicol 95, 95–103 32428649 10.1016/j.reprotox.2020.05.006 PMC7323850

[63] Rosner B (2011): Fundamentals of biostatistics. Seventh edition. Boston : Brooks/Cole, Cengage Learning, [2011] ©2011

[64] Sánchez Brisa N, Hu H, Litman Heather J, Téllez-Rojo Martha M (2011): Statistical Methods to Study Timing of Vulnerability with Sparsely Sampled Data on Environmental Toxicants. Environ Health Perspect 119, 409–415 21362588 10.1289/ehp.1002453 PMC3060007

[65] Sanyal T, Bhattacharjee P, Bhattacharjee S, Bhattacharjee P (2018): Hypomethylation of mitochondrial D-loop and ND6 with increased mitochondrial DNA copy number in the arsenic-exposed population. Toxicology 408, 54–61 29940200 10.1016/j.tox.2018.06.012

[66] Shadel Gerald S, Horvath Tamas L (2015): Mitochondrial ROS Signaling in Organismal Homeostasis. Cell 163, 560–569 26496603 10.1016/j.cell.2015.10.001 PMC4634671

[67] Smith Anna R, Lin Pi ID, Rifas-Shiman Sheryl L, Rahman Mohammad L, Gold Diane R, Baccarelli Andrea A, Claus Henn B, Amarasiriwardena C, Wright Robert O, Coull B, Hivert M-F, Oken E, Cardenas A (2021): Prospective Associations of Early Pregnancy Metal Mixtures with Mitochondria DNA Copy Number and Telomere Length in Maternal and Cord Blood. Environ Health Perspect 129, 117007 34797165 10.1289/EHP9294 PMC8604047

[68] Soler-Blasco R, Murcia M, Lozano M, Sarzo B, Esplugues A, Vioque J, Lertxundi N, Marina LS, Lertxundi A, Irizar A, Braeuer S, Goesler W, Ballester F, Llop S (2021): Urinary arsenic species and methylation efficiency during pregnancy: Concentrations and associated factors in Spanish pregnant women. Environ Res 196, 110889 33607098 10.1016/j.envres.2021.110889

[69] Soler-Blasco R, Murcia M, Lozano M, Sarzo B, Esplugues A, Riutort-Mayol G, Vioque J, Lertxundi N, Santa Marina L, Lertxundi A, Irizar A, Braeuer S, Ballester F, Llop S (2022): Prenatal arsenic exposure, arsenic methylation efficiency, and neuropsychological development among preschool children in a Spanish birth cohort. Environ Res 207, 112208 34662579 10.1016/j.envres.2021.112208

[70] Song L, Liu B, Wang L, Wu M, Zhang L, Liu Y, Bi J, Yang S, Zhang B, Xia W, Xu S, Chen R, Cao Z, Wang Y (2020): Exposure to arsenic during pregnancy and newborn mitochondrial DNA copy number: A birth cohort study in Wuhan, China. Chemosphere 243, 125335 31765894 10.1016/j.chemosphere.2019.125335

[71] Stajnko A, Šlejkovec Z, Mazej D, France-Štiglic A, BriŠKi AS, Prpić I, Špirić Z, Horvat M, Falnoga I (2019): Arsenic metabolites; selenium; and AS3MT, MTHFR, AQP4, AQP9, SELENOP, INMT, and MT2A polymorphisms in Croatian-Slovenian population from PHIME-CROME study. Environ Res 170, 301–319 30612060 10.1016/j.envres.2018.11.045

[72] Steinmaus C, Yuan Y, Kalman D, Atallah R, Smith AH (2005): Intraindividual Variability in Arsenic Methylation in a U.S. Population. Cancer Epidemiol Biomarkers Prev 14, 919–924 15824164 10.1158/1055-9965.EPI-04-0277

[73] Steinmaus C, Ferreccio C, Acevedo J, Balmes JR, Liaw J, Troncoso P, Dauphiné DC, Nardone A, Smith AH (2016): High risks of lung disease associated with early-life and moderate lifetime arsenic exposure in northern Chile. Toxicol Appl Pharmacol 313, 10–15 27725189 10.1016/j.taap.2016.10.006 PMC5247272

[74] Stýblo M, Drobná Z, Jaspers I, Lin S, Thomas David J (2002): The role of biomethylation in toxicity and carcinogenicity of arsenic: a research update. Environ Health Perspect 110, 767–771 12426129 10.1289/ehp.110-1241242 PMC1241242

[75] Tian X, Wang M, Ying X, Dong N, Li M, Feng J, Zhao Y, Zhao Q, Tian F, Li B, Zhang W, Qiu Y, Yan X (2023): Co-exposure to arsenic and fluoride to explore the interactive effect on oxidative stress and autophagy in myocardial tissue and cell. Ecotoxicol Environ Saf 253, 114647 36801539 10.1016/j.ecoenv.2023.114647

[76] Urata M, Koga-Wada Y, Kayamori Y, Kang D (2008): Platelet contamination causes large variation as well as overestimation of mitochondrial DNA content of peripheral blood mononuclear cells. Ann Clin Biochem 45, 513–514 18753426 10.1258/acb.2008.008008

[77] Vahter M (2002): Mechanisms of arsenic biotransformation. Toxicology 181-182, 211–217 12505313 10.1016/s0300-483x(02)00285-8

[78] Valko M, Morris H, Cronin TDM (2005): Metals, Toxicity and Oxidative Stress. Curr Med Chem 12, 1161–1208 15892631 10.2174/0929867053764635

[79] Vriens A, Nawrot TS, Baeyens W, Den Hond E, Bruckers L, Covaci A, Croes K, De Craemer S, Govarts E, Lambrechts N, Loots I, Nelen V, Peusens M, De Henauw S, Schoeters G, Plusquin M (2017): Neonatal exposure to environmental pollutants and placental mitochondrial DNA content: A multi-pollutant approach. Environ Int 106, 60–68 28600986 10.1016/j.envint.2017.05.022

[80] Wai T, Langer T (2016): Mitochondrial Dynamics and Metabolic Regulation. Trends Endocrinol Metab 27, 105–117 26754340 10.1016/j.tem.2015.12.001

[81] Wang X, Wu Y, Sun X, Guo Q, Xia W, Wu Y, Li J, Xu S, Li Y (2021): Arsenic exposure and metabolism in relation to blood pressure changes in pregnant women. Ecotoxicol Environ Saf 222, 112527 34311426 10.1016/j.ecoenv.2021.112527

[82] Wei Y-H, Lee C-F, Lee H-C, Ma Y-S, Wang C-W, Lu C-Y, Pang C-Y (2001): Increases of Mitochondrial Mass and Mitochondrial Genome in Association with Enhanced Oxidative Stress in Human Cells Harboring 4,977 BP-Deleted Mitochondrial DNA. Ann N Y Acad Sci 928, 97–112 11795533 10.1111/j.1749-6632.2001.tb05640.x

[83] Wei Y-H, Lee H-C (2002): Oxidative Stress, Mitochondrial DNA Mutation, and Impairment of Antioxidant Enzymes in Aging. Exp Biol Med (Maywood) 227, 671–682 12324649 10.1177/153537020222700901

[84] Wu M, Shu Y, Song L, Liu B, Zhang L, Wang L, Liu Y, Bi J, Xiong C, Cao Z, Xu S, Xia W, Li Y, Wang Y (2019): Prenatal exposure to thallium is associated with decreased mitochondrial DNA copy number in newborns: Evidence from a birth cohort study. Environ Int 129, 470–477 31158593 10.1016/j.envint.2019.05.053

[85] Yakes FM, Van Houten B (1997): Mitochondrial DNA damage is more extensive and persists longer than nuclear DNA damage in human cells following oxidative stress. Proc Natl Acad Sci U S A 94, 514–519 9012815 10.1073/pnas.94.2.514 PMC19544

